# Supplementary material for: Altered microRNA Transcriptome in Cultured Human Liver Cells upon Infection with Ebola Virus
Source: Int J Mol Sci. 2021 Apr 6;22(7):3792. doi: 10.3390/ijms22073792 (PMC8038836; doi:10.3390/ijms22073792)
Supplement: Supplementary file 1 [file ijms-22-03792-s001.zip › Supplementary_File/C_ GO_Analysis_Results/16-30nt_go_Reston-24h-Huh7_vs_Control-24h-Huh7_down.mature_mirna_targets/CC_FoldEnrichment.pdf]

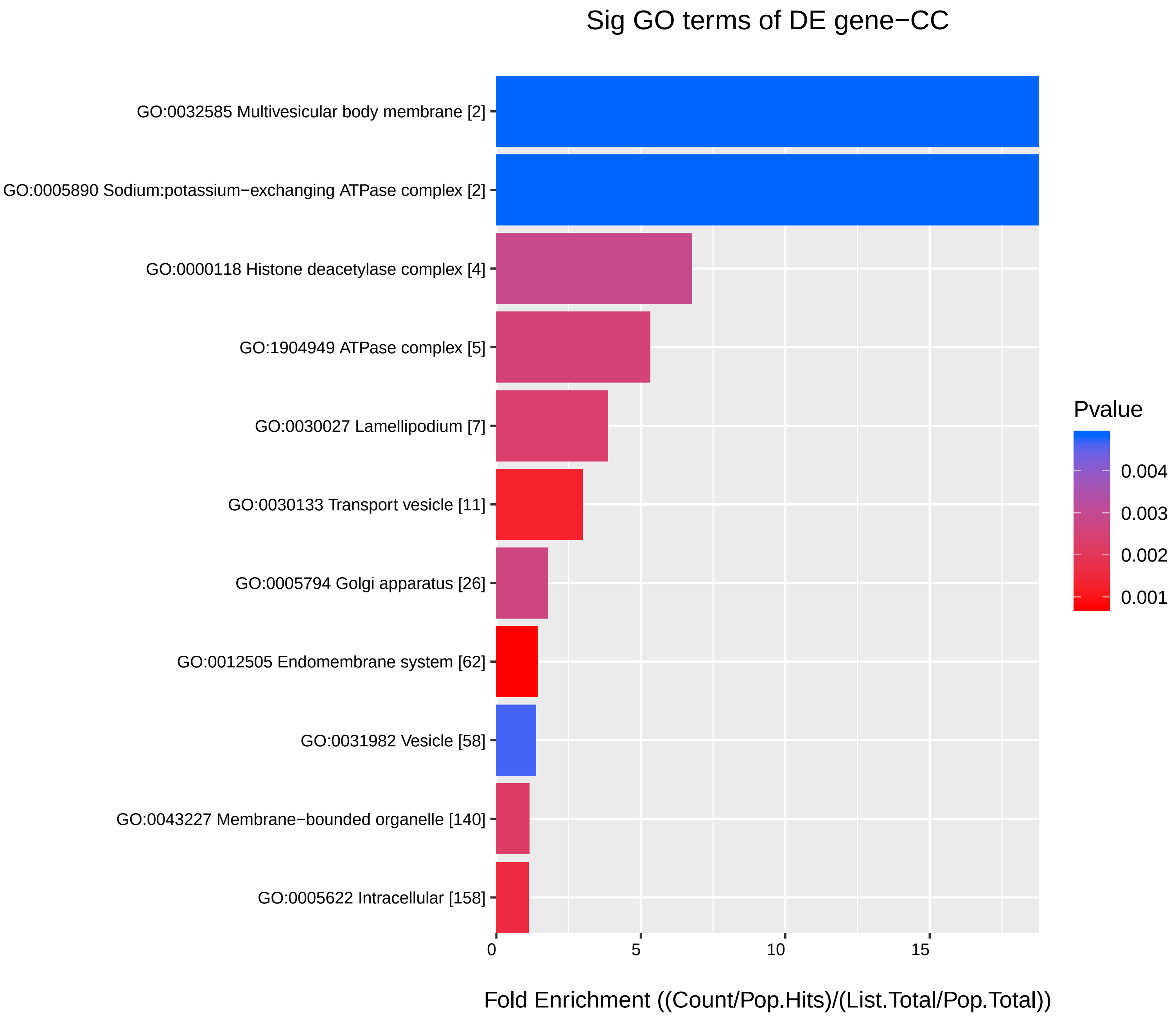
Sig GO terms of DE gene−CC
GO:0032585 Multivesicular body membrane [2]
GO:0005890 Sodium:potassium−exchanging ATPase complex [2]
GO:0000118 Histone deacetylase complex [4]
GO:1904949 ATPase complex [5]
GO:0030027 Lamellipodium [7]
GO:0030133 Transport vesicle [11]
GO:0005794 Golgi apparatus [26]
GO:0012505 Endomembrane system [62]
GO:0031982 Vesicle [58]
GO:0043227 Membrane−bounded organelle [140]
GO:0005622 Intracellular [158]
0
5
10
15
Fold Enrichment ((Count/Pop.Hits)/(List.Total/Pop.Total))
Pvalue
0.004
0.003
0.002
0.001
